# Pulse amplitude ratio under noninvasive ventilation as a new method in the diagnosis of left heart failure in patients with acute exacerbation of chronic obstructive pulmonary disease

**DOI:** 10.1186/s12872-023-03089-y

**Published:** 2023-02-24

**Authors:** Khaoula Bel Haj Ali, Adel Sekma, Ikram Chamtouri, Kaouthar Beltaief, Mohamed Amine Msolli, Zied Mezgar, Wahid Bouida, Riadh Boukef, Hamdi Boubaker, Mohamed Habib Grissa, Semir Nouira

**Affiliations:** 1 Emergency Department, Fattouma Bourguiba University Hospital, 5000 Monastir, Tunisia; 2 Research Laboratory LR12SP18, University of Monastir, 5019 Monastir, Tunisia; 3 Cardiology Department, Fattouma Bourguiba University Hospital, 5000 Monastir, Tunisia; 4 Emergency Department, Farhat Hached University Hospital, 4031 Sousse, Tunisia; 5 Emergency Department, Sahloul University Hospital, 4011 Sousse, Tunisia

**Keywords:** Non-invasive ventilation, Chronic obstructive pulmonary disease, Heart failure, Pulse amplitude ratio

## Abstract

**Background:**

Left heart failure (LHF) is commonly associated with acute exacerbation of chronic obstructive pulmonary disease (AECOPD) but its role is often underestimated.

**Aim of study:**

To evaluate the performance of a new diagnostic technique based on the measurement of the pulse amplitude ratio (PAR) using non-invasive ventilation (NIV) for the early identification LHF in patients admitted to the emergency department (ED) for AECOPD.

**Results:**

73 patients were included in this study: 32 in LHF group and 41 in non LHF- group. The two groups had comparable demographic and clinical characteristics at admission. The mean values of PAR_NIV_ was significantly higher among LHF patients (0.86 vs. 0.71; *p* < 0.01). The area under the receiver operating characteristic curve of PAR_NIV_ was 0.75. Using the best cut-off (0.6), the sensitivity of PAR_NIV_ was 93% with a specificity 21%, a positive predictive value of 48%, and a negative predictive value of 81%. Correlation between PAR_NIV_ and BNP was significant (r = 0.52; *p* = 0.002).

**Conclusion:**

Measurement of PAR_NIV_ in patients presenting to the ED with AECOPD had a good diagnostic performance for the detection of LHF and could represent an interesting alternative for the currently available methods.

*Trial registration* The study was registered in the Clinical Trial Registration System (clinicaltrials.gov) under the study number NCT05189119, https://register.clinicaltrials.gov/prs/app/action/SelectProtocol?sid=S000BOO4&selectaction=Edit&uid=U0000QAM&ts=2&cx=qrmluh.

## Introduction

Chronic obstructive pulmonary disease (COPD) is a universally prevalent chronic disease. It is a major public health problem due to its increasing prevalence, high morbidity and mortality rates [1]. On average, a COPD patient suffers one to four exacerbations per year. The origin of these exacerbations is not easy to determine, but most often a viral or bacterial infection is incriminated. Left heart failure (LHF) is commonly associated with acute exacerbations of COPD (AECOPD), but this association is frequently underestimated [2–5]. Symptoms and signs of LHF are particularly difficult to identify and interpret, especially in obese and elderly subjects [4, 6–9]. Therefore, the use of additional investigation methods, such as echocardiography and invasive hemodynamic exploration, is decisive for the diagnosis, but it cannot be considered in the emergency room on a routine basis [10]. The tendency to generalize cardiac ultrasound is still limited by its dependency on the operator skills and experience [11, 12]. Brain Natriuretic Peptide (BNP) is now widely used for the diagnosis of LHF [13]. However, a BNP value in the grey zone does not allow the determination of the etiology of dyspnea; this is the case for approximately 30% of patients consulting the emergency room for dyspnea [14]. In addition, elevated BNP levels are observed in the elderly, patients with renal failure, chronic pulmonary disease, hypoxemia and right ventricular congestion [15]. Alternative diagnostic method, based on the effect of Valsalva maneuver (VM) on blood pressure, has been proposed [16]. VM consists of forced exhalation with a closed glottis, which leads to an increase in intra thoracic pressures, the effect of which on the blood pressure curve depends on whether there is or not LHF. Several studies have shown that this maneuver could identify patients with LHF through the measurement of the pulse amplitude ratio (PAR) [17–19]. Recently, Boubaker et al. found that VM could be used to identify LHF in patients with AECOPD [20]. However, VM is sometimes difficult to be correctly performed for dyspneic patients. Thus, we propose, in this study, an alternative method to VM. It consists in applying a positive pressure through NIV with inspiratory support. We aimed to evaluate the value of a new method of measuring PAR based on the application of pressure support ventilation (PSV) to assist in the early diagnosis of LHF in patients presenting to the ED with AECOPD.

## Patients and methods

This is an observational cross-sectional study of patients who presented to the ED of Fattouma Bourguiba University Hospital (Tunisia) with acute dyspnea in the context of AECOPD. The study protocol has been prepared in accordance with the revised Helsinki Declaration for Biomedical Research Involving Human Subjects and Guidelines for Good Clinical Practice. The study was approved by Fattouma Bourguiba ethics committee under the number IOG009738 N°90; the patient’s informed consent was obtained before the start of the protocol. The study was registered in the Clinical Trial Registration System (clinicaltrials.gov) under the study number NCT05189119 on the 12/01/2022.

### Study population

#### Inclusion criteria

Adult patients with a history of COPD who consulted the emergency department for moderate to severe AECOPD defined as acute worsening of respiratory symptoms that result in additional therapy were included [2]. COPD is defined according to the criteria of the American Thoracic Society as a progressive airflow limitation that is not fully reversible associated with a chronic inflammatory response of the lungs to noxious particles or gases [21].

#### Exclusion criteria

Patients with hemodynamic instability (cardiogenic shock, myocardial infarction), coma and severe hypoxemia (SpO_2_ < 60%) or acidosis (pH < 7.1) were excluded. Patients with contraindications to NIV (Glasgow score < 12, swallowing disorder, severe bronchial obstruction, massive retention of secretions despite bronchoscopy, vomiting, those with anatomical or functional upper airway obstruction or ongoing upper gastrointestinal bleeding or ileus), non-cooperative patients as well as those who refused to give consent were also excluded.

### Methodology

**Fig. 1:**
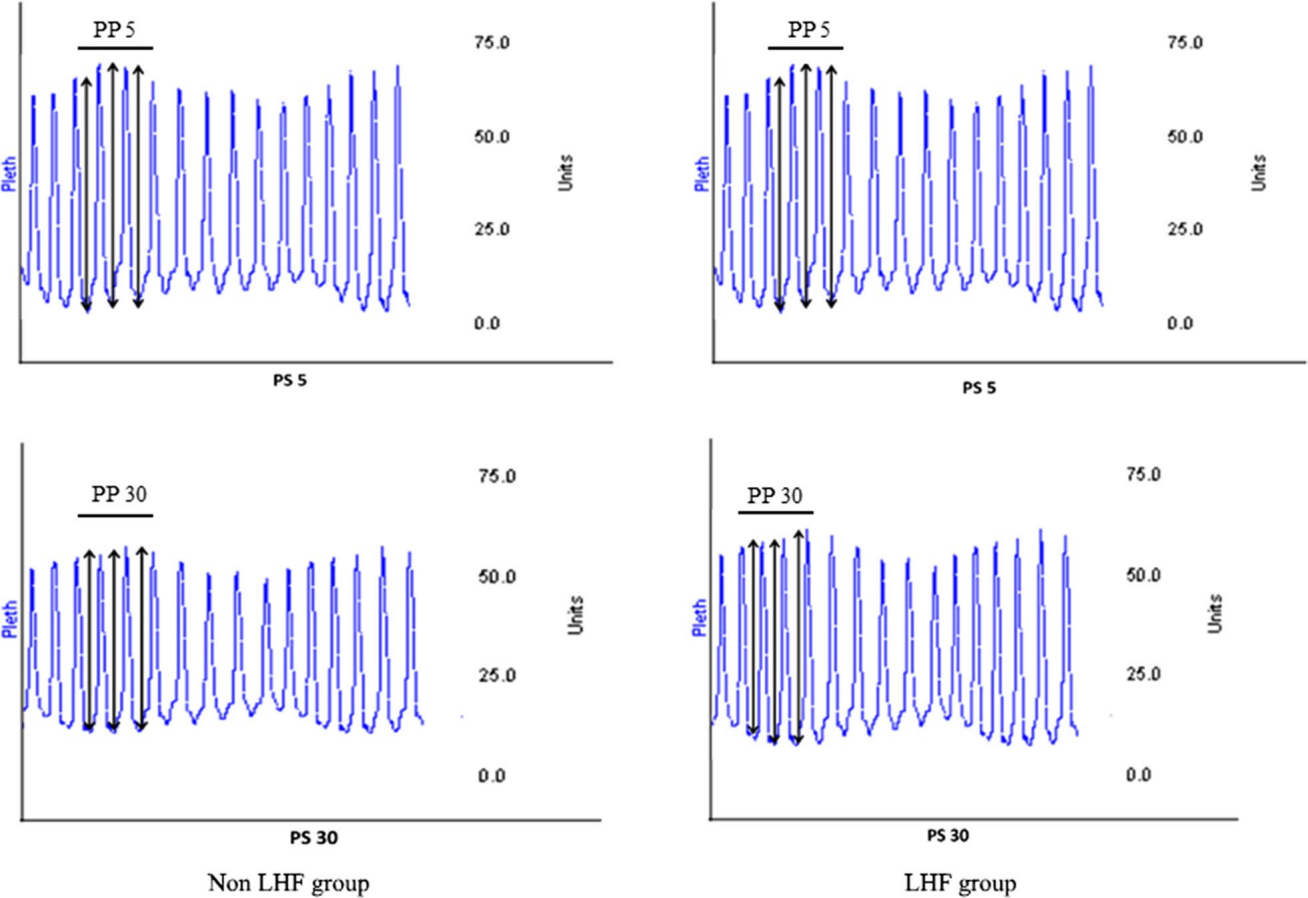
Measurement of pulse amplitude ratio using plethysmographic blood pressure signal under non-invasive ventilation with two levels of pressure support (5 and 30 cmH_2_O). *LHF* left heart failure, *PP5* average of 3 consecutives pulse pressure values under pressure support of 5 cmH_2_O. *PP30* average of 3 consecutive pulse pressure values under pressure support of 30 cmH_2_O

Patients’ demographic characteristics, medical histories, number of COPD exacerbations during the last 12 months, smoking and current drug treatment, as well as clinical examination data including blood pressure, heart rate, respiratory rate, pulse oxygen saturation, and temperature were collected. Complementary examination findings were also recorded (electrocardiogram, chest X-ray, cardiac ultrasound, BNP, blood gas, and standard biological tests). Patients were prepared for monitoring with three-lead electrode ECG and a plethysmographic sensor on the finger to obtain two simultaneous records. The plethysmographic PAR was calculated using a data acquisition and analysis device (BIOPAC Systems, CA, USA). Each enrolled patient received NIV applied through an oro-nasal mask with a positive end expiratory pressure of 4 cmH_2_O, inspiratory oxygen fraction ensuring pulse oxygen saturation of 88–92%, and inspiratory pressure support (PS) set at 5 and 30 cmH_2_O. The 2 PS levels (5 and 30 cmH_2_O) were investigated for 15 min in random order, with 10 min spontaneous breathing in between the ventilation periods to return to baseline level. Three successive measurements of PAR during inspiration time were taken 5 min after each level of pressure support onset. In all case, leak should always be minimized by mask adjustment and/or by changing the mask type. The following calculations were performed: PP5 which is the average of the plethysmographic pulse pressure measured during three inspiratory cycles recorded under PS = 5 cmH_2_O. PP30 which is the average of the plethysmographic pulse pressure measured during three inspiratory cycles recorded under PS = 30 cmH_2_O (Fig. 1). We defined PAR_NIV_ as the PP30/PP5 ratio. All the measurements were performed by the same investigators who were not aware of the clinical and biological details of the patient. The treating physicians were blinded to the results. The patients were divided into two groups: a group of patients having LHF (LHF group) and a group of patients without LHF (non LHF group). The diagnosis of LHF was made by two experts on the basis of clinical data, elevated natriuretic peptides levels (BNP > 100 pg/ml) and cardiac ultrasound findings [left ventricular systolic dysfunction with reduced LVEF (40% or less) or preserved ejection fraction with elevated estimated LV filling pressure (E/e′ ratio > 9)] [22]. In case of a disagreement, a third senior physician was consulted and adjudicated the case.

### Statistical analysis

Qualitative data were expressed with frequencies and percentage. Chi-square test or Fisher exact test were used to compare qualitative variables as appropriate. Normality was assessed with the Shapiro–Wilk test for continuous variables. Data are presented as mean (standard deviation) or median (interquartile range) for continuous variables according to their distribution. Comparison of data between the groups was performed using a two-sample Student’s t-test or non-parametric tests, as appropriate. We calculated the sensitivity, specificity, negative predictive value and positive predictive value of PAR_NIV_ using the best cut-off. The choice of the PAR threshold was based on the level having the best sensitivity and specificity on the ROC curve (Youden index). A *p* value less than 0.05 was statistically significant. The correlation between PAR_NIV_ and BNP and between PAR_NIV_ and left ventricular ejection fraction (LVEF) was assessed. All data were analyzed using the SPSS version 20.0 statistical software.

## Results

**Fig. 2:**
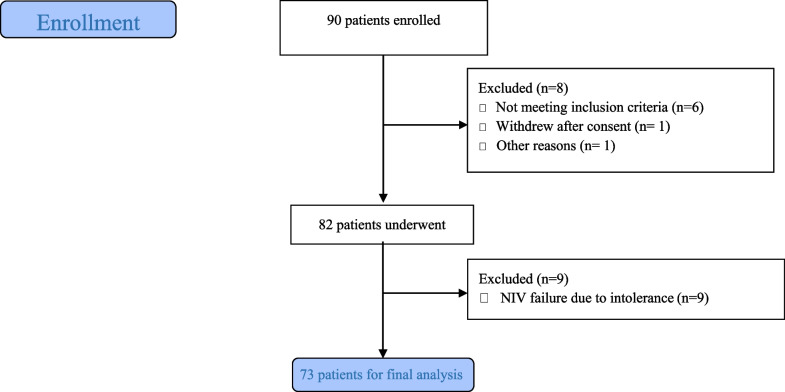
Flow chart

**Fig. 3:**
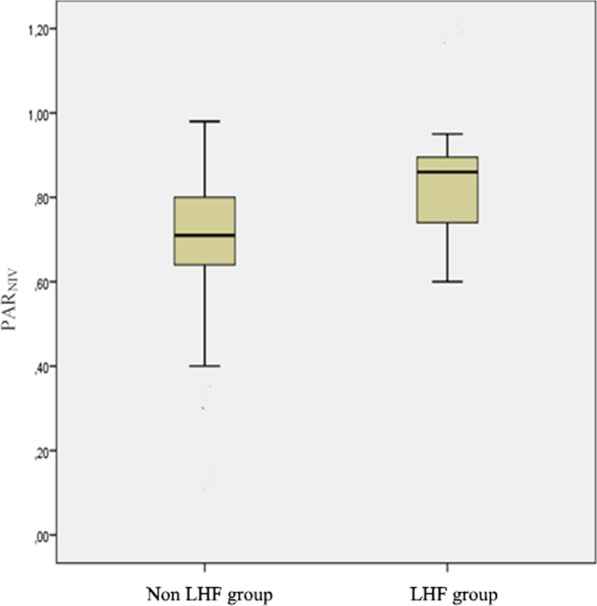
Pulse amplitude ratio under non-invasive ventilation in patients with left heart failure (LHF group PAR_NIV_) and patients without left heart failure (non LHF group). *PAR*_*NIV*_ pulse amplitude ratio under non-invasive ventilation, *LHF* left heart failure

**Fig. 4:**
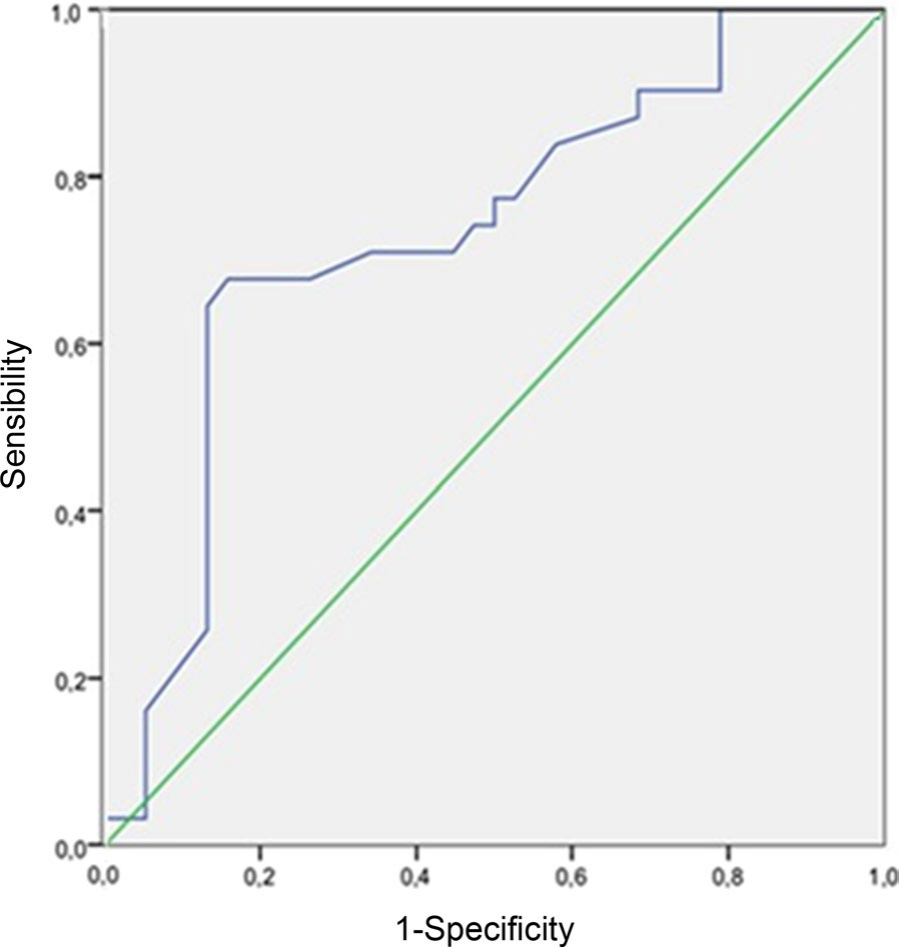
Area under the ROC curve of pulse amplitude ratio under non-invasive ventilation. *PAR*_*NIV*_ pulse amplitude ratio under non-invasive ventilation, *AUC* area under the ROC curve

**Fig. 5:**
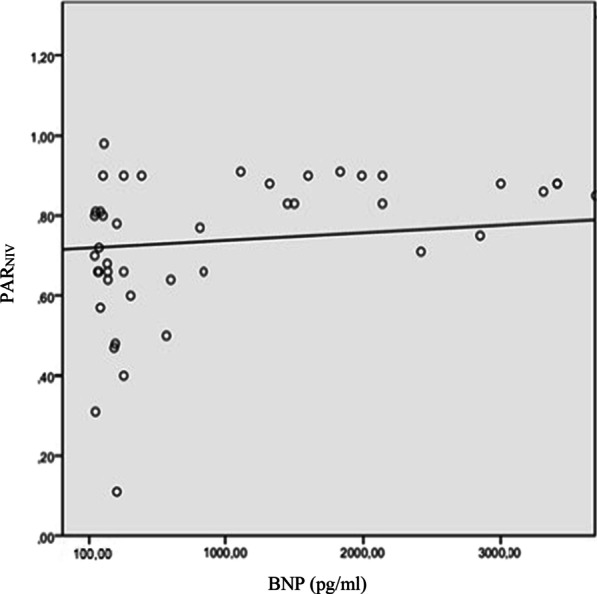
Correlation between pulse amplitude ratio under non-invasive ventilation and BNP values. *PAR*_*NIV*_ pulse amplitude ratio under non-invasive ventilation, *BNP* brain natriuretic peptide

**Fig. 6:**
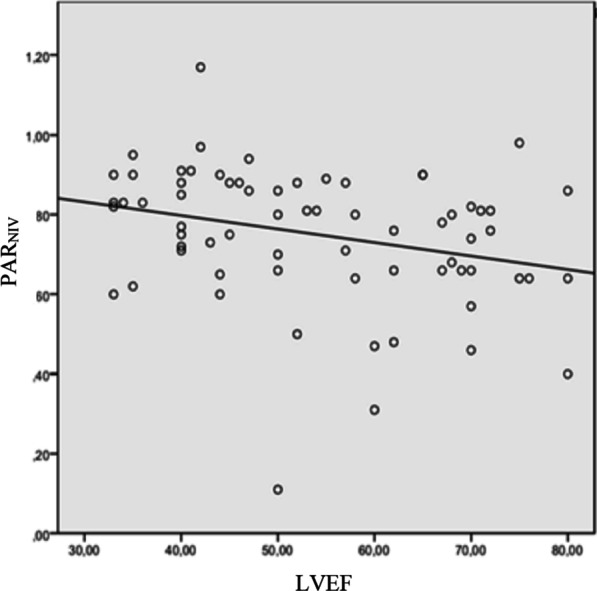
Correlation between Pulse amplitude ratio under non-invasive ventilation and left ventricular ejection fraction values. *PAR*_*NIV*_ pulse amplitude ratio under non-invasive ventilation, *LVEF* left ventricular ejection fraction

Over the 5-month study period extending from January, 03, 2022 to May, 03, 2022, 73 patients were included, among whom 32 had a confirmed diagnosis of LHF (Fig. 2). Table 1 summarizes the characteristics of the overall population. The mean age was 67 ± 9 years for the LHF group and 68 ± 10 years for the non LHF group. A male predominance was observed in both groups, with 26 male patients (81%) in the LHF group and 36 patients (87%) in the non LHF group. Previous history of hypertension was found in 46% of patients and coronary artery disease in 25% in the LHF group. This proportion represented 34% and 10%, in the non LHF group with no statistically significant difference. Mean BNP values were 195 ± 190 pg/ml in the non LHF group patients and 3421 ± 1200 pg/ml in the LHF group (*p* < 0.001). LVEF was 43.9 ± 11% and 60 ± 13.5 in the LHF and non LHF groups respectively (*p* < 0.001). Mean PAR values were 0.86 ± 0.12 in the LHF group and 0.71 ± 0.14 in the non LHF group (*p* < 0.01) (Fig. 3).The area under the ROC curve for PAR_NIV_ was 0.75 with the best threshold at PAR_NIV_ = 0.6 (Fig. 4). Using this cut-off, the sensitivity was 93%, specificity 21%, positive predictive value 48%, and negative predictive value 81%. The correlation between PAR_NIV_ and BNP was significant (r = 0.52; *p* = 0.002) (Fig. 5). PAR_NIV_ was inversely correlated with LVEF (r = − 0.337; *p* = 0.005) (Fig. 6).

**Table 1:** Patients’ baseline characteristics

	LHF group n = 32	Non LHF group n = 41	*p*
Age years, mean ± SD	67 ± 9	68 ± 10	0.87
Sex-ratio M/F	26/6	36/5	0.44
Smoking (pack-years), mean (SD)	42.8 (15.9)	44.6 (15.7)	0.85
Body mass index (kg/m^2^), mean (SD)	26.5 (4.3)	26.5 (5.8)	0.91
Exacerbations within the past year, mean (SD)	2.4 (1.5)	2.1 (0.9)	0.75
Past medical history, n (%)			
Chronic heart failure	12 (37)	2 (4)	**< 0.001**
Coronary artery disease	8 (25)	4 (10)	0.08
Hypertension	15 (46)	14 (34)	0.27
Diabetes	8 (25)	9 (21)	0.75
Atrial fibrillation	7 (22)	3 (7)	0.14
Clinical parameters, mean ± SD			
SBP (mmHg)	137 ± 23	142 ± 32	0.42
DBP (mmHg)	72 ± 17	79 ± 22	0.58
Respiratory rate (c/min)	29 ± 8	27 ± 7	0.77
Heart rate (b/min)	103 ± 16	106 ± 24	0.82
Temperature (°C), mean (SD)	37.1 (0.5)	37.1 (0.6)	0.97
Biology			
pH, median (IQR)	7.33 (0.04)	7.32 (0.05)	0.94
PaCO_2_ (KPa), median (IQR)	6,03 (2.2)	7,01 (1)	0.63
PaO_2_ (KPa), median (IQR)	9,1 (3.2)	8,8(3)	0.65
HCO_3_^−^ (mmol/l), mean ± SD	24 ± 5	25 ± 4	0.73
SaO_2_ (%), mean ± SD	88 ± 6	82 ± 9	0.66
BNP(pg/ml), mean ± SD	3421 ± 1200	195 ± 190	**< 0.001**
LVEF (%), mean ± SD	43.9 ± 11	60 ± 13.5	**< 0.001**
E/A, median, IQR	1.5 (1.5)	1.2 (1.1)	**< 0.001**
E/e′, median, IQR	12.2 (6.8)	9 (4)	**< 0.001**

Bold values indicate* p* < 0.05

*LVEF* left ventricular ejection fraction, *E/A* late to early mitral diastolic Doppler velocity ratio, *M/F* male/female, *SD* standard deviation, *IQR* interquartile range, *SBP* systolic blood pressure, *DBP* diastolic blood pressure, *PaCO*_*2*_ partial pressure of carbon dioxide, *PaO*_*2*_ partial pressure of oxygen, *HCO*_*3*_^−^ serum bicarbonate level, *SaO*_*2*_ arterial oxygen saturation, *BNP* brain natriuretic peptide

## Discussion

In our study, we proposed a new method using NIV as a mean of LHF diagnosis during AECOPD. We found the following main results: (1) The PAR_NIV_ was higher in the LHF group compared to the non LHF group. (2) There was a correlation between the PAR_NIV_ value and BNP as well as between the PAR_NIV_ and LVEF. (3) Using the best cut-off (0.6) the PAR_NIV_ had a good sensitivity (93%). LHF and COPD are two common conditions in clinical practice. Both conditions share common risk factors, such as advanced age, smoking, and systemic inflammation which explain their frequent coexistence. Among the co-morbidities most commonly associated with COPD, congestive LHF is the most difficult to diagnose leading to a potential detrimental delay in the management of patient’s condition, or on the contrary, to unnecessary treatment [6]. LHF diagnostic uncertainty can be as high as 40% [23]. Chest X-ray and ECG are easily accessible diagnostic tests, but their value in identifying LHF is limited in COPD patients [4, 9, 24]. BNP values, as isolated markers of LHF, are sometimes insufficient given that a number of non-cardiac conditions may be associated with BNP elevation [13]. Diagnosing LHF without echocardiography is often challenging and the best diagnostic approach remains unclear. Besides, echocardiography is not always accessible in the ED, and measurements depend on the operator experience. Moreover, the poor acoustic window caused by pulmonary air trapping may hinder accurate echocardiographic assessment of LHF in 10–35% of COPD patients, particularly in those with more severe degrees of airflow obstruction [24]. The study of the effect of intra-thoracic pressure variation on the cardiovascular system is likely to provide insight into cardiac function and help in the early and rapid diagnosis of LHF. This principle has been applied in numerous studies for the identification of left ventricular dysfunction. One of the most recent studies was conducted by Remmen et al. in which VM and hemodynamic exploration by a Swan-Ganz catheter were performed [25]. The results showed a very good correlation between pulmonary capillary wedge (pressure PCWP) and PAR (r = 0.63; *p* < 0.001). The area under the ROC curve of PAR for the diagnosis of HF (PCWP > 15 mmHg) was 0.85. This correlation still remains valid even in patients with arrhythmias. Similar results were found in the study of Wilemann et al. who performed invasive hemodynamic exploration by right catheterization and analyzed the blood pressure response to VM in 42 patients in a stable state [26]. They found a very strong correlation between PCWP and PAR (r = 0.75; *p* < 0.001). In addition, treatment-induced changes in PCWP correlated well with changes in PAR (r = 0.93; *p* < 0.001. McIntyre et al. found similar results when they replaced VM with the application of intrathoracic positive pressure using airway occlusion under continuous positive airway pressure [27]. In mechanically ventilated patients, Zema et al. showed that an abnormal response to VM was associated with a simultaneous increase in central venous pressure and PCWP [28]. The rectangular shape of blood pressure record under VM was consistently associated with an increase in PCWP within a range of 20–37 mmHg. In a recent study, Boubaker et al. showed that plethysmographic PAR during VM was higher in patients with AECOPD with left ventricular dysfunction compared to those without left ventricular dysfunction (0.78 vs. 0.38, *p* = 0.01). Similarly, they reported that PAR value was not significantly different between COPD group with left ventricular dysfunction and a third group with heart failure and no COPD diagnosis (*p* = 0.78). The area under the ROC curve for plethysmographic PAR to detect left ventricular dysfunction was significantly higher compared to that of the Boston score (0.92 vs. 0.76; *p* = 0.02) [20]. In practice, VM is difficult to apply in COPD patients in respiratory distress. Therefore, in our study, we proposed the application of NIV using two levels of PS (5 and 30 cmH_2_O) to simulate the effects of VM. To our knowledge, this is the first study to use this method as a diagnostic tool of LHF in AECOPD patients.

Our study has some limitations. First, we recognize the small sample size of our study conducted on an ED population for whom it is not easy to perform such extensive explorations. Second, we used the plethysmographic signal instead of the invasive blood pressure records. It is true that this signal can be modified by artifacts, but it was shown that the respiratory variations of the invasive and non-invasive blood pressure values were correlated [29]. Third, the level of PS that should be used must be sufficient to create the intrathoracic pressure necessary to induce an effect on the cardiovascular system. We have used a level of 30 cmH_2_0 because beyond this level there is a risk of increasing leakage through the respiratory circuit and abdominal distension. In COPD patients, this PS level could be considered as high with regard to hyperinflation and intrinsic PEEP often present in this population. Hence, our method should be used with caution in AECOPD patients and possibly after measuring intrinsic PEEP. Although we did not report any adverse events related to barotrauma or any patient discomfort during the protocol, it is prudent to avoid PAR_NIV_ testing in patients with high baseline intrinsic PEEP. Finally, our technique does not allow to affirm the role of LHF in the actual exacerbation episode of COPD.

In conclusion, the PAR_NIV_ measurement could become in the future a useful index to facilitate the diagnosis of LHF in AECOPD patients. Our results should be confirmed on a larger sample of patients.

## Data Availability

The datasets generated and/or analysed during the current study are not publicly available but are available from the corresponding author on reasonable request.

## Abbreviations

HF: Heart failure
COPD: Chronic obstructive pulmonary disease
AECOPD: Acute exacerbation of chronic obstructive pulmonary disease
PAR: Pulse amplitude ratio
NIV: Non-invasive ventilation
ED: Emergency department
PS: Pressure support
PP: Plethesmographic pulse pressure
AUC: Area under roc curve
BNP: Brain natriuretic peptide
VM: Valsalva maneuver
PSV: Pressure support ventilation
LVEF: Left ventricular ejection fraction
PCPW: Pulmonary capillary wedge pressure
